# Calorie Restriction Effects on Aging, Learning Performance, and Transcription in Aged *Aplysia californica*

**DOI:** 10.1111/gbb.70046

**Published:** 2026-02-26

**Authors:** Eric C. Randolph, Lynne A. Fieber

**Affiliations:** ^1^ Department of Marine Biology and Ecology University of Miami Rosenstiel School Miami Florida USA; ^2^ Department of Biology University of Alabama at Birmingham Birmingham Alabama USA

**Keywords:** aging, *Aplysia californica*, calorie restriction, learning food is inedible, RNA‐sequencing

## Abstract

Along with increasing an animal's lifespan, calorie restriction (CR) is shown to improve an animal's cognition. To elucidate the molecular differences that accompany CR that may benefit cognition, sibling *Aplysia californica* were reared on either an ad‐lib (AL) or CR diet. Siblings from each diet were trained in two behaviors, learning food is inedible (LFI) and habituation of the tail withdrawal reflex (TWR), at two time points along their lifespans: younger animals at training time 1 (TT1) and aged siblings at training time 2 (TT2). In analysis by diet, TT2 CR animals' learning performance was on par with their TT1 CR siblings in both paradigms, illustrating a maintenance of cognition in age. Meanwhile, TT2 AL animals performed worse than TT1 AL siblings in habituation but better in LFI, illustrating the lack of cognitive maintenance in age. RNA sequencing was performed on part of the buccal ganglia that houses many of the neurons involved in LFI. Gene expression results implied morphological changes occurring within the motor and interneurons of the buccal ganglia after learning in LFI. These neurons showed enrichment of *protein kinase C binding* (GO:0005080), *cadherin binding* (GO:0045296), and *microtubule severing ATPase activity* (GO:0008568) as well as *neuroactive ligand‐receptor interaction* (ko04080) and *valine, leucine and isoleucine biosynthesis* (ko00290) all of which have been implicated to assist in memory consolidation and reconsolidation.

## Graphical abstract

Buccal ganglia inter‐ and motoneuronal transcriptional changes were investigated after learning food is inedible (LFI) in *Aplysia californica* reared on different diets at two ages. Those reared on calorie restriction showed delayed signs of aging and maintained their performance in LFI when aged while those reared on ad‐lib did not.

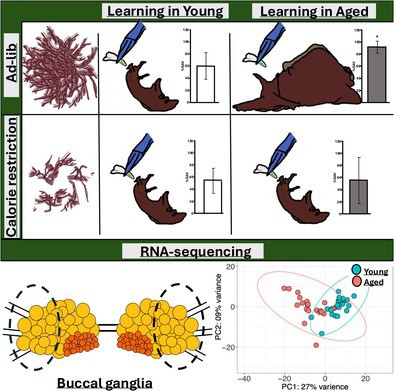

## 1 Introduction

Calorie restriction (CR) has been the most successful experimental intervention to increase lifespan, demonstrated in yeast [1], *Caenorhabditis elegans* [2], *Drosophila* [3], rats [4], some primates [5], and other animals [6]. Accompanying lifespan extension are a reduction in body fat [7], reduced metabolic rate [8], attenuation of oxidative damage [9], enhancement or inhibition of apoptosis [10, 11], increased autophagy [12], reduction of inflammation [13], increased physical activity [14], reduced plasma glucose levels [15], reduced or increased IGF1‐ [16, 17] and insulin signaling [18, 19], reduced TOR signaling [20], and hormesis [21, 22]. Some of these auxiliary effects may themselves increase lifespan [23]. Currently unclear is whether CR initiates physiological effects that facilitate lifespan extension, or whether it is acting to slow biological aging.

Biological aging is regarded as distinct from chronological aging [24, 25, 26]. While every living being experiences the same passage of time, individuals experience frailty and loss of fitness at different rates [25]. Such changes are shown to be better predictors of the onset of mortality [27] and many age‐related diseases [28]. Biological aging can be measured via molecular biomarkers such as DNA methylation [27, 29, 30], or physical fitness indicators such as grip strength [31] and gait speed [32, 33]. In the neural model *Aplysia californica* (Aplysia), biological aging is measurable via reflex behaviors in known‐age animals in a hatchery setting [34].

Less studied is the concept that CR increases learning performance, addressed in aged rodents [35, 36, 37] and some human trials [38]. The gains in learning performance in CR corresponded with increases in ionotropic glutamate receptors for N‐methyl‐D‐aspartate (NMDA) and alpha‐amino‐3‐hydroxy‐5‐methyl‐4‐isoxazolepropionic acid (AMPA) [35, 36], the mitigation of neuron loss [39], enhanced synaptic efficacy [36], and increased expression of neural genes such as brain‐derived neurotrophic factor (BDNF), insulin‐like growth factor 1, adenosine monophosphate‐activated protein kinase (AMPK), and glucose transporter type 4 (GLUT4) [40, 41]. Like CR effects on lifespan extension, it is unclear whether CR facilitates physiological changes that enhance learning performance in age, or if CR slows brain aging. Other open questions include whether there are CR effects on synaptic efficacy and neuron retention at any age [42, 43, 44, 45].

Tested here is whether cognitive performance gains occur in chronologically older vs. younger sibling Aplysia in which CR has delayed biological aging. There were no signs of cognitive gains or losses in older CR Aplysia, suggesting maintenance of cognition in age. This was compared to the multiple signs of cognitive changes in older Aplysia fed ad‐lib (AL). Gene expression results largely support these ideas.

## 2 Materials and Methods

### 2.1 Animal Husbandry and Reflex Behavior

A cohort of simultaneous hermaphroditic *Aplysia californica* (Aplysia) from wild‐caught parents was raised in the University of Miami's National Aplysia Resource. All animals were siblings from the same egg mass. At 5 months post‐hatch, animals were placed into four holding cages at a density of 100 per cage. Two of the holding cages, totaling 200 animals, received nominally ad‐lib (AL) rations as described in Fieber et al. [46], where *Agardhiella subulata* was weighed before feeding, and after 4 h little to no food was left in the cage. The remaining holding cages, totaling 200 animals, received calorie restriction (CR) rations at 60% of AL. 2 weeks later, *Ulva lactuca* was incorporated into the diets of both treatments, at 90% *A. subulata* and 10% *U. lactuca* .

At age 6 months 45 AL and 45 CR animals were transferred to exercise regimes as described in Fieber et al. [46] where an overhead dump bucket forcefully ejected water into each cage every 5–7 min to mimic the animal's natural habitat. Transferred animals were weighed and then placed in cages as groups of five according to mass. Aplysia were selected visually for their size before being transferred, excluding any exceptionally large or small animals, keeping animals of similar weights together. The standard deviation of weights within groups remained less than 7.4 g. Animals remained in exercise regimes until either sampled as controls, tested in LFI then sampled, or recorded natural lifespan.

Animals' biological age stage was determined by the significant increase in time to complete two reflex behaviors, the righting reflex (TTR) and the tail withdrawal reflex (TWR) as described in Kempsell and Fieber [34]. Reflex behaviors were measured monthly in at least 13 haphazardly chosen animals from each diet treatment at ages 9–11 months. By age 12 months, most animals had been sampled, leaving eight AL and five CR animals for testing reflex behaviors.

### 2.2 CR Determination

Once animals were placed in exercise regimes, CR rations were adjusted to 65% total weight of all five animals per cage per week, divided over 6 days. Standard hatchery rations at the National Aplysia Resource were previously established to be 90% of an animal's body weight per week [47]. CR rations aimed to be less than this. AL animals were supplied with constant food. Animals were weighed weekly, and CR rations were calculated for the coming week. Growth curves for all AL and CR animals were recorded.

To ensure CR was established without incurring malnutrition, the CR growth curve was closely monitored. Failure to gain weight or the loss of weight within the first few months on CR before LFI training began were considered signs of malnutrition. This was not observed. CR animals were also assessed daily for any behaviors that have been previously attributed to distress or poor health due to food deprivation such as spontaneous inking, loss of the ability to adhere to the cage surface with the foot, or flaring of the parapodia and exposure of the gills [48]. These behaviors were also not observed. All CR animals were normal in appearance and behavior.

In addition, since malnutrition affects the way organs and muscles develop, proximate analyses and fatty acid profiles were obtained for foot muscle tissue from AL and CR animals to investigate any indications of malnutrition in CR animals that could not be assessed visually. These tests have been previously used to assess the effects of malnutrition on developing piglet intestines [49] and were used here to assess any drastic changes in Aplysia muscle composition in animals reared on CR. Two samples of ~50 g of pooled foot muscle tissue were obtained from four AL animals. This allowed for duplicate testing in AL. Due to the significantly smaller size of the CR animals, 11 CR animals were sampled to obtain one ~50 g sample of pooled foot muscle tissue. Samples were sent to Midwest Laboratories for analysis. Results from the two AL samples were averaged and then compared to the result from the one CR sample. Any observation of similarities to Lopez‐Pedrosa et al. [49], or any large differences in fatty acid profiles of the Aplysia muscle tissues would be considered an indication that CR animals were malnourished.

### 2.3 Learning Food Is Inedible

Animals were trained in learning food is inedible (LFI) at two training times, TT1 and TT2, intended to compare learning performance along their lifespan. Learning performance during these training times was reconciled with animals' stages of aging based on reflex behavior according to Kempsell and Fieber [34]. For TT1, LFI training was conducted at ages 7–9 months. All TT1 AL animals were sexually mature (M stage) at the beginning of TT1 while CR siblings transitioned to sexual maturity toward the end of TT1 training, at age 9 months, likely due to their much smaller size. TT2 occurred when animals were 10 months old. AL animals were in stage AII [34], the most advanced age stage, and CR animals were in stage M.

The specific protocol for LFI in this study was adapted from Susswein et al. [50] and described in detail in Randolph and Fieber [51]. In brief, a probe of netted *U. lactuca* held in a plastic hemostat was presented to hungry animals that had been fasted for 48 h. The probe was held about 1 cm in front of the animals' oral tentacles and the animals were allowed to advance toward the probe and initiate their biting reflex through tactile stimulation of the animals' lips by the probe. Every time the probe was ejected from the animals' oral cavity it was repositioned to 1 cm in front of the animals' oral tentacles and the process was repeated. Two researchers were required for this protocol, one who administered the training and another who took notes. Upon presentation of the probe, the note taker started a pair of stopwatches and privately noted the times that corresponded to specific behaviors that were observed by the researcher holding the probe. These behaviors were each time the animal took the probe into its mouth, each time the animal ejected the probe from its mouth, whenever the animal's radula could be felt scraping the probe through vibrations of the hemostat, and when 3 min elapsed from an ejection of the probe from the mouth. The latter was the criterion for the time training ended for that day. A total time in the mouth (TTIM) was calculated by adding the time the animal held the probe in its mouth during each ingestion attempt. If TTIM was > 100 s the animal was placed in a holding tank with a non‐experimental companion for 26 h, with no food, when Day 2 of LFI recall testing began. Animals that did not attain a TTIM > 100 s were labeled “dud” and excluded from further testing and analysis. Day 2 of LFI followed the same protocol as Day 1.

On Day 2 of LFI, animals were expected to attain a TTIM < Day 1's performance; however, the experimenter was unaware of Day 1 animal performance. The percentage of total time saved relearning inedibility of the probe on Day 2 compared to Day 1 (%SAV) was used as a measure of how well that animal learned [52, 53] and was calculated using the following equation,

$$\%\mathrm{SAV}=\left(1-\frac{\mathrm{Day}\;2\;\text{TTIM}}{\mathrm{Day}\;1\;\text{TTIM}}\right)\times 100$$

Animals that attained a Day 1 TTIM > 100 s and subsequently a Day 2 TTIM < Day 1 TTIM achieved a positive %SAV (+%SAV) were considered to have trained successfully, and demonstrated a long‐term memory (LTM) of LFI. Only +%SAV animals were analyzed for behavioral comparisons, habituation of TWR, and gene expression. Animals that attained a Day 2 TTIM > Day 1 TTIM had a negative %SAV (−%SAV), were considered to not have demonstrated an LTM of LFI, and were excluded from further testing and analysis consistent with Tam et al. [54]. Thus, excluded samples were −%SAV animals and duds.

Training in LFI continued in each training time until at least six animals from each diet condition attained a + %SAV. Both AL and CR animals were trained in LFI on the same day, alternating between the two. Animals with +%SAV were sacrificed 2 h after Day 2 LFI recall testing. Untrained sibling animals were sacrificed as controls for gene expression analyses.

### 2.4 Habituation of Tail Withdrawal Reflex

Animals that achieved +%SAV in LFI were subjected to habituation training of TWR as previously described in Kempsell and Fieber [55]. Animals were placed in a 16 L plastic cage filled with aerated seawater and allowed to acclimate for 10 min. The animal's length was measured, and its tail was depressed to half its thickness with the end of a piece of 125 lb. test fishing line for 1 s, causing the animal to contract its tail. The animal's contracted length was then measured. TWR amplitude was determined by expressing the animal's contracted length as a fraction of its initial length. The TWR duration was also recorded as the time it took from initiation of contraction to when the animal relaxed its tail to 30% of its pre‐contracted length. A baseline for each animal was established by taking the average of three tail taps with interstimulus intervals (ISI) of 5 min starting at 15, 10, and 5 min before habituation training began (pre‐test). Habituation training consisted of 50 TWR episodes at 30 s ISI which was followed by three post‐habituation episodes at 5, 10, and 15 min after training (post‐test).

### 2.5 Statistical Analysis

The Shapiro–Wilk test was used to test for normality in all data collected before appropriate statistical tests were chosen for that dataset. Differences in reflex behavior times over the lifespan of the animals within each diet group were assessed using the Kruskal‐Wallis rank sum tests post hoc Dunn test (*p* ≤ 0.05). Differences in reflex behavior times within each month of testing between the two diet groups were assessed using multiple Wilcoxon rank sum tests followed by Bonferroni multiple test correction (padj ≤ 0.0125). Significant differences each week in animal weights between the two diet groups were assessed via Two‐Way ANOVA post hoc Tukey (*p* ≤ 0.05). Lifespan differences between diets were assessed using a Kaplan–Meier survival analysis.

Differences in %SAV, TTIM, the number of radular scrapes of the probe (bite frequency), the amount of time it took animals to complete LFI training (total elapsed time; TET), and TWR habituation amplitude were assessed by Wilcoxon Signed Rank, Student's, or paired *T*‐tests when appropriate and often followed by a Bonferroni. Specifics on which tests were used for which datasets are detailed in the Results.

### 2.6 RNAseq

Buccal ganglia of two sibling groups were prepared for RNASeq: trained animals that achieved a + %SAV, which were sacrificed 2 h after Day 2 recall testing in LFI, and untrained control animals, sacrificed after a 76‐h fast (to equal the duration of fasting in the LFI‐tested animals). Animals were anesthetized by injecting chilled magnesium chloride at a volume of 1/6 body mass into the posterior sinus. Buccal ganglia were dissected out, rinsed with artificial seawater (ASW; NaCl, MgCl_2_, CaCl_2_, KCl, HEPES, pH 7.6–7.8, 980 mosM), and pinned to the sylgard base of a glass Petri dish containing ASW. Under a dissecting microscope, the buccal ganglia were trimmed as detailed in Randolph and Fieber [51]. Conspicuous neurons B1 and B2 were located under the dissecting microscope and the buccal ganglia were cut along a vertical axis to the side of B1 nearest the buccal sensory neuron clusters (BSCs), eliminating the BSCs and many inter‐ and motoneurons from trimmed samples.

Trimmed buccal ganglia were placed into 1.5 mL microcentrifuge tubes containing 300 μL RNAProtect (Qiagen). Tubes were incubated at 4°C for 24 h and stored at −80°C in preparation for RNA extraction. Total RNA was extracted using the RNeasy Micro Kit (Qiagen) following the manufacturer protocol and the recommended 15‐min DNA digestion step with DNase1 (Qiagen). RNA was eluted in RNase‐free water. Total RNA quality and concentration were determined using a Nanodrop (Technologies Model ND‐1000).

Library preparation and sequencing of total RNA samples was carried out at the John P. Hussman Institute for Human Genomics (HIHG) at the University of Miami's Miller School of Medicine. Prior to library prep, total RNA quality and quantity for all samples were confirmed on an Agilent Tapestation. Illumina sequencing libraries were constructed from total RNA using the Nugen Ovation SoLo RNA‐Seq library preparation kit following the manufacturer protocol. Libraries were polyA selected and sequenced with an Illumina NovaSeq 6000 at a requested depth of 30 million paired‐end 100 base pair reads per sample.

Raw read files were checked for quality with the software FastQC [56]. Adaptors were trimmed from raw read sequences with BBDuk software from the BBtools package [57]. FastQC indicated that all raw reads were of sufficient quality and that no additional trimming was necessary prior to alignment. Trimmed reads were aligned to the Aplysia reference genome (AplCal3.0 GCF 000002075.1) using STAR software [58]. Aligned reads were quantified using Salmon [59]. A transcript database (TxDb) was constructed from the Aplysia reference Gene Transfer Format file (gtf) via the *R* package GenomicFeatures [60] in R [61]. Tximport [62] was then used to import transcript abundances into the *R* statistical environment. Transcript abundances were imported to the gene level and to the transcript level for two different analyses. For the first analysis on differential gene expression, transcript abundances were summarized to the gene level and converted to counts using the tximport option “tx2gene” and the generated TxDB transcript to gene object generated from the reference genome. A final filtering of gene counts was then undertaken, with genes that had less than one total count in at least four samples removed from downstream analyses. For the second analysis of differential transcript usage, transcript abundances were not summarized to gene level and scaled up to library size.

### 2.7 Principal Component and Differential Gene Expression Analyses

DESeq2 [63] was used to run a principal component analysis (PCA). Gene counts were first normalized via variance stabilizing transformation (VST) and then used as input for the PCA. It was noticed that multiple samples fell outside the 95% confidence interval (CI) of their respective clusters and were assumed to be outliers. CIs were then raised to 99.9% to increase confidence in visually determining outliers, and samples that were still outside of this new CI were assumed to be thus. A secondary robust principal component analysis (rPCA) using rrcov::PcaGrid [64] was then performed for statistical verification [65]. Samples that were determined outliers in the rPCA were removed from the metadata, the transcript abundances of the remaining samples were then re‐imported into *R* using tximport, filtered to remove any genes that had less than one total count in at least four samples, normalized via VST, and used as input for a second PCA.

The variance of each principal component (PC) of the PCA was then determined using PCAtools [66] and illustrated using the “screeplot” function, taking note of the number of PCs necessary for 70% of the variance to be accounted for. The “eigencorplot” function of PCAtools was used to correlate the eigenvalue of PC1–PC12 to the experimental independent variables.

DESeq2 was used for differential expression analysis. The original formula that was input for DESeq2's statistical model included just the three independent variables (factors) in this experiment which were chronological age (Age), learning food is inedible (LFI), and diet (Diet). Each factor consisted of two levels which were Age: TT1 or TT2; LFI: untrained (U) or trained (T); and Diet: AL or CR. After the statistical model was constructed a fourth independent variable was added to account for complete interactions of all other factor levels, resulting in 8 interaction terms (Figure S1). Thus, an interaction term (group level) labeled TT2UCR refers to animals that are at a chronological age which corresponds to TT2, untrained in LFI, and reared on a CR diet. This was done to precisely investigate the effects when only one variable was altered at a time. This method of statistical model construction was consistent with the DESeq2 vignette. A Wald test was then conducted to detect differentially expressed genes (DEG) using a contrast of the complete interaction terms just mentioned. Genes with an adjusted *p*‐value ≤ 0.05 and a lfcThreshold = 0 were considered significantly differentially expressed. Six contrasts were run in total and DEGs from each contrast were compared.

For clarity, contrasts between interaction terms were given sensible nomenclature. “Age AL” refers to chronologically older TT2UAL vs. chronologically younger TT1UAL, “Age CR” refers to TT2UCR vs. TT1UCR, “LFI young AL” refers to TT1TAL vs. TT1UAL, “LFI aged AL” refers to TT2TAL vs. TT2UAL, “LFI young CR” refers to TT1TCR vs. TT1UCR, and “LFI aged CR” refers to TT2TCR vs. TT2UCR (Table S1). Each contrast relates to how genes or functional analysis terms change in the first group compared to the second. Thus, an increase in chronologically older (TT2) AL samples is compared to chronologically younger (TT1) AL samples.

### 2.8 Hierarchical Clustering Analysis

All DEGs from the six contrasts were used in a hierarchical clustering analysis to view how samples clustered according to their independent variables. First, a regularized log transformation was applied to DEG read counts. Then, transformed counts were scaled around one. Finally, clustering was accomplished using pheatmap [67].

### 2.9 Gene Ontology and Kyoto Encyclopedia of Genes and Genomes Enrichment Analysis

After the six contrasts were run in DESeq2 for each comparison of interaction terms, the results were sorted in descending order of log2 fold change. The *R* package clusterProfiler [68] was used to perform a gene set enrichment analysis (GSEA) on the sorted results to identify significantly enriched gene ontology (GO) terms. Due to Aplysia not having an org.db profile and clusterProfiler requiring one, a *de‐novo* org.db from NCBI annotations was constructed using AnnotationForge::makeOrgPackageFromNCBI [69]. To increase reproducibility and account for random seed setting in the algorithm, the seed value was set to “1234” for all GSEA analyses and the argument seed = TRUE was added when running clusterProfiler::gseGO. Significant GO terms were filtered by qvalue to remove any significant terms with qvalues over 0.80.

To identify enriched Kyoto Encyclopedia of Genes and Genomes (KEGG) pathways, the Aplysia proteome was uploaded to GhostKOALA [70] and protein‐specific k numbers were assigned to each protein in the Aplysia proteome based on their amino acid sequences. Proteins were then mapped back to their genes and k numbers were assigned to each gene within the log2 fold change sorted results mentioned above. A GSEA was performed using clusterProfiler::gseKEGG with the option keyType = “kegg”, organism = “ko”, and seed = TRUE to identify enriched KEGG pathways. Significant KEGG pathways were filtered by qvalue to remove any significant pathways with qvalues over 0.80.

### 2.10 Differential Transcript Usage

Differential transcript usage (DTU) was investigated by not summarizing to gene level when importing counts into *R* and using the tximport option “txOut = TRUE” and “countsFromAbundance = ‘scaledTPM’”. Following the import of transcript abundances with tximport, DTU was assessed following the protocol outlined in Love et al. [71]. First, the package DRIMSeq [72] was used to filter transcripts and identify genes that showed evidence of DTU. Transcripts were filtered to eliminate those that had less than one count in at least four samples, had a relative abundance proportion less than 1%, or mapped to a corresponding gene that had less than one total count in four or less samples. Then the results from DRIMSeq were fed into stageR [73] to control for the overall false discovery rate.

## 3 Results

### 3.1 Aging Staging for Learning Food Is Inedible From Reflex Behaviors

**FIGURE 1:**
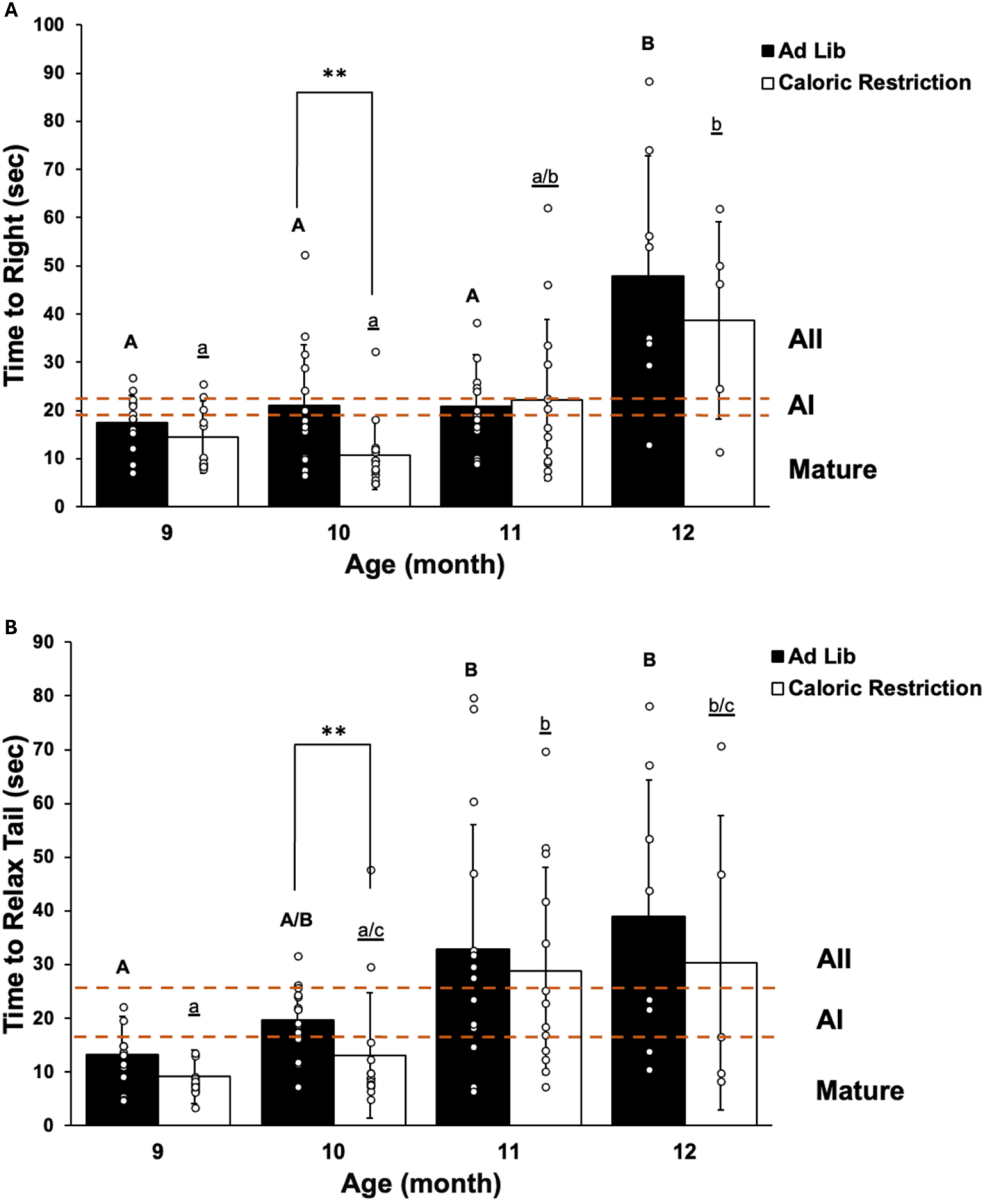
Reflex behaviors for the cohort. (A) Mean time to right in the righting reflex, ±SD. (B) Mean time to relax tail in the tail withdrawal reflex, ±SD. Dashed lines indicate cutoff times to complete reflexes in the Aplysia stages of aging [34] with the corresponding stage at right. Changes in letters above bars indicate significance differences via Kruskal‐Wallis rank sum tests with post hoc Dunn test (*p* ≤ 0.05) in reflex completion times between months within a diet group, with capital letters for AL animals and lowercase, underlined letters for CR animals. **denotes a significant difference between diet groups within the same month via Wilcoxon rank sum tests followed by Bonferroni multiple test correction (padj ≤ 0.0125). *n* = 13 for each month of assessment except at 12 months when 8 AL and 5 CR animals were tested.

Ad‐lib (AL) animals became sexually mature at age 7 months, just prior to the beginning of training time TT1 of learning food is inedible (LFI), and thus were designated Mature (M) via the Aplysia stages of aging [34]. Reflex behavior testing of time to right (TTR) and tail withdrawal reflex (TWR), shown in Figure 1, began at age 9 months, when AL animals were in transition from M to stage AI based on their reflex completion times (Figure 1A,B). AL animals spent only a brief period in AI, transitioning to stage AII at age 10 months when training time TT2 of LFI began.

Calorie restriction (CR) animals transitioned from sexual immaturity to maturity during the TT1 LFI training period. Figure 1A,B show CR animals remained in stage M through age 10 months, and thus although they were the same chronological age, they were two age stages younger than AL animals during TT2 LFI training. This significant delay in CR behavioral aging (TTR: Wilcoxon, *p* ≤ 0.05; TWR: Wilcoxon, *p* ≤ 0.05) lasted 1 month, as all animals in the experiment advanced to stage AII at age 11 months.

### 3.2 Life and Death

**FIGURE 2:**
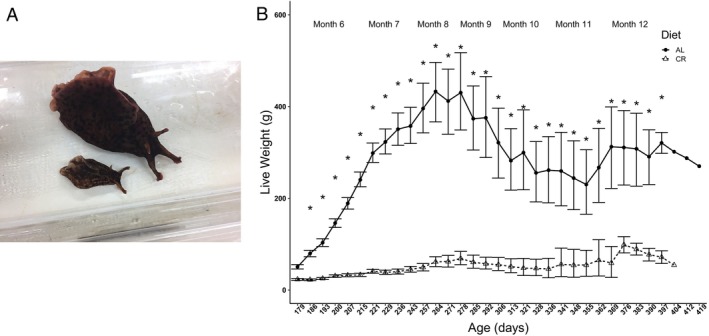
(A) Sibling animals at training time point TT1, age 7 months, on AL (top) and CR (bottom) diets. (B) Growth curves for each diet group starting on the day animals were placed in exercise regimes at 6 months post‐hatch. Initial data points are the mean weight of all 45 animals in that diet group ±SD. Subsequent data points began to decrease in sample size as animals were sacrificed following LFI (beginning at age 7 months) or died at the end of their natural lifespan (beginning at ~315 days). *denotes significance difference in animal weights between the two diets at that age via Two‐Way ANOVA post hoc Tukey *p* ≤ 0.05.

**FIGURE 3:**
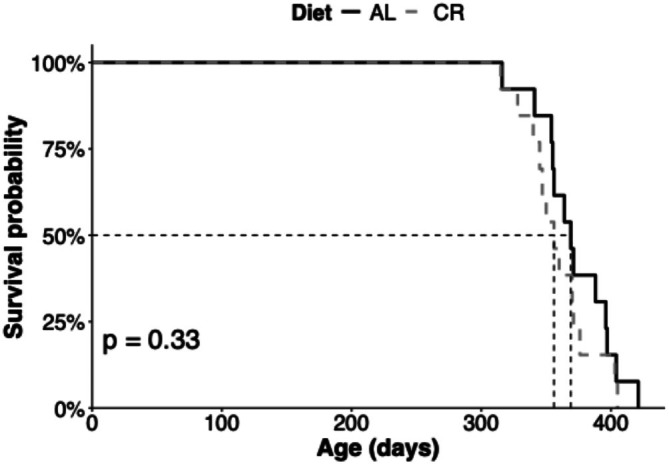
Survival functions for the cohort separated by diet (*n* = 16 for both diets). A Kaplan–Meier test indicated no difference in median survival between animals reared on the two diets.

AL animals weighed significantly more than their CR siblings at each weekly weigh‐in from the beginning of the experiment at age 6 months (two‐way ANOVA, *p* ≤ 0.05, *F* = 22.72 (age), *F* = 2325.03 (diet), *F* = 17.21 (age: diet); Tukey, *p* ≤ 0.05) (Figure 2A,B). The profile of the growth curves was similar for each diet. CR did not significantly affect lifespan in this study (Kaplan–Meier, *p* = 0.33) (Figure 3). The first natural animal deaths occurred on day 315, and by day 404 there were too few animals in each diet group to statistically test for differences in weights. The final CR animal died 16 days before the final AL animal, at ages 13 and 13.5 months, respectively.

### 3.3 Nutrient Analysis

Chemical analysis results of Aplysia foot muscle tissue tested for possible malnutrition in the CR group are presented in Tables S2–S5. Differences between the two diet groups were minute or absent (Tables S2 and S3), except for one odd chain saturated fatty acid C17:0, 10‐heptadecanoic acid, which was reduced by 7% in CR foot muscle. CR animals also contained less saturated and polyunsaturated fats while having higher monosaturated fats than their AL siblings (Tables S4 and S5). All chemical values from CR animals were either within the standard deviation range of AL samples or fell < 1.5% outside of that range (saturated fats).

### 3.4 Learning Food Is Inedible

AL and CR animals were at different stages of aging at the TT1 and TT2 training times for LFI, corresponding to chronological ages 7–9 and 10 months, respectively. There were two types of learning failures during LFI training: failure to achieve a TTIM > 100 s on Day 1 (duds), and a successful Day 1 training followed by a regression where Day 2 TTIM > Day 1 TTIM resulting in a negative percent savings (−%SAV). Successful TT1 LFI training was achieved in six AL and six CR animals. Ten additional AL were duds at TT1, and one CR had −%SAV. Successful TT2 LFI training occurred in five AL and six CR animals, with one −%SAV AL, eight AL duds, one CR −%SAV, and four CR duds. All subsequent results represent only the +%SAV animals and their same‐aged untrained siblings, as in Table 1.

**TABLE 1:** Final LFI and differential expression (DE) sample sizes for each category. Numbers in parentheses are revised DE sample sizes after removal of outliers.

Diet/training time	LFI trained	Untrained control
Calorie restriction		
TT1	6	6 (4)
TT2	6	6
Ad‐Lib		
TT1	6	6
TT2	5	6

Sample sizes for this study were purposely kept small for several reasons. First, LFI is a well‐established protocol that has been performed regularly with consistent results in Aplysia since 1983 [74] and in *Aplysia californica* since 2008, specifically showing 100 s TTIM is sufficient to produce LTM [75]. There is also an example of using a reduced 50 s TTIM criterion [76]. Additionally, sample sizes were kept low so that RNA‐sequencing could be performed on every animal that successfully completed LFI and demonstrated a LTM. This was designed to avoid any bias in subsampling a larger behavioral sample size for molecular assays. An *N* = 6 was chosen according to Schurch et al. [77], where it was stated that six biological replicate samples per condition are sufficient for RNA‐sequencing when using DESeq2, with more than six resulting in diminishing returns. Our lab previously used a larger sample size in LFI [51]. We found the behavioral results from the two studies were consistent.

**FIGURE 4:**
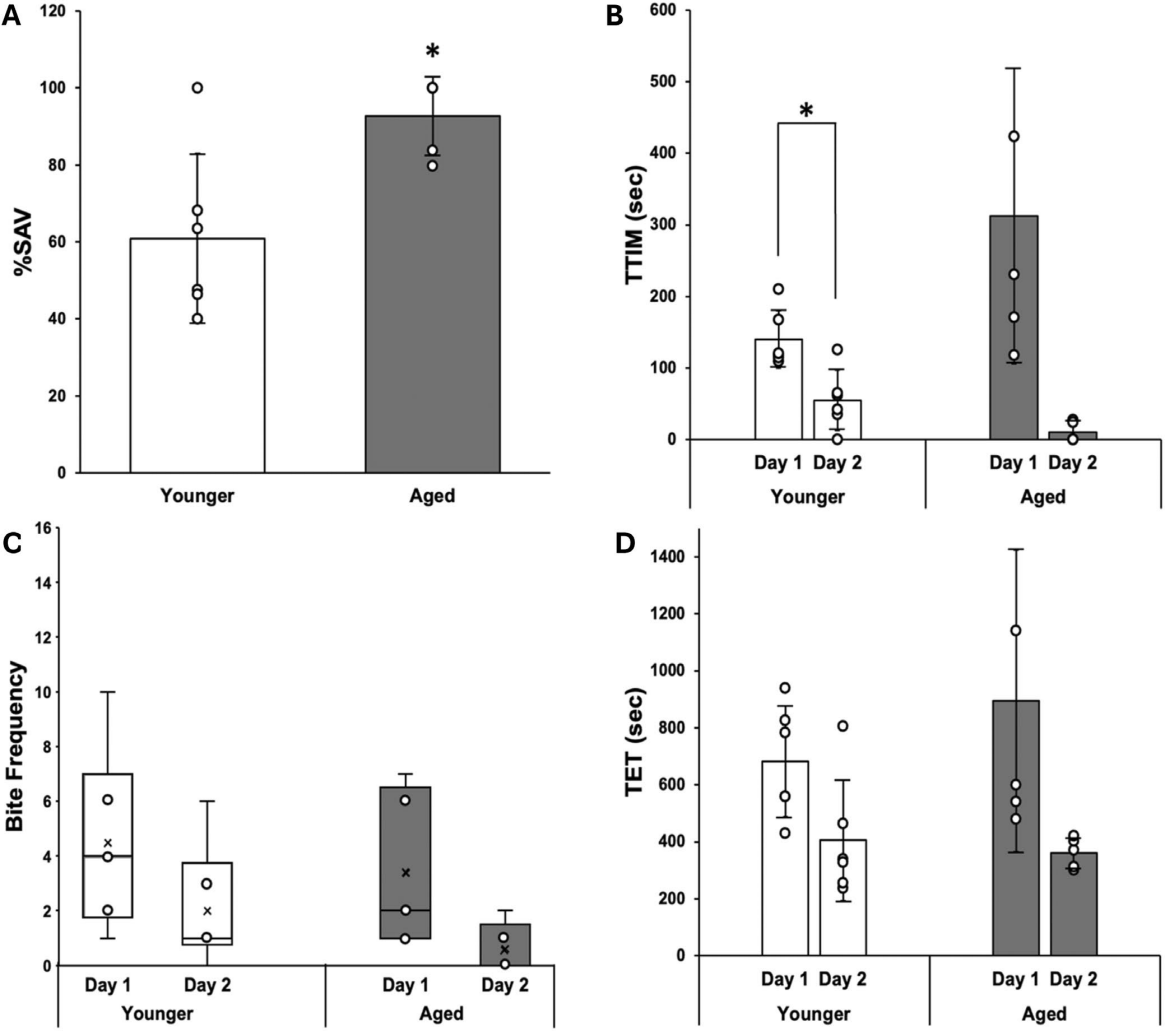
LFI metrics in AL sibling Aplysia. (A) Mean %SAV, ±SD. *denotes significant difference via Student's T‐test; *p* ≤ 0.05 (*p* = 0.0152). (B) Mean TTIM, ±SD, on Day 1 and Day 2 training. *denotes significant difference via Wilcoxon Signed‐Rank test; *p* ≤ 0.05 (younger *p* = 0.03552). (C) Distribution of ingestion attempts (bite frequency) on Day 1 and Day 2 of LFI. (D) Mean total elapsed time (TET) to completion of LFI for each day of training.

LFI performances for both TT1 and TT2 AL animals (Figure 4A–D) were identical to those observed in Randolph and Fieber [51], with faster probe recognition on Day 2 in metrics such as total time in mouth (TTIM) (TT1: Wilcoxon, *p* ≤ 0.05), bite frequency, and total elapsed time (TET). Additionally, TT2 AL animals achieved a significantly higher %SAV than their TT1 AL siblings (*t*(7) = 3.16, *p* ≤ 0.05) (Figure 4A), which was also consistent with the earlier study. Of note was the phenomenon of TT2 animals ignoring the probe altogether on Day 2. Of the five TT2 AL animals that attained a + %SAV, three achieved 100% SAV by not attempting to ingest the food probe at all on Day 2. These three animals had a two‐fold higher Day 1 TTIM than the other TT2 AL trainees (Figure 4B), which resulted in the high standard deviation (SD) for TTIM and an absence of difference in this metric between Day 1 and Day 2 (Wilcoxon, *p* = 0.0625).

**FIGURE 5:**
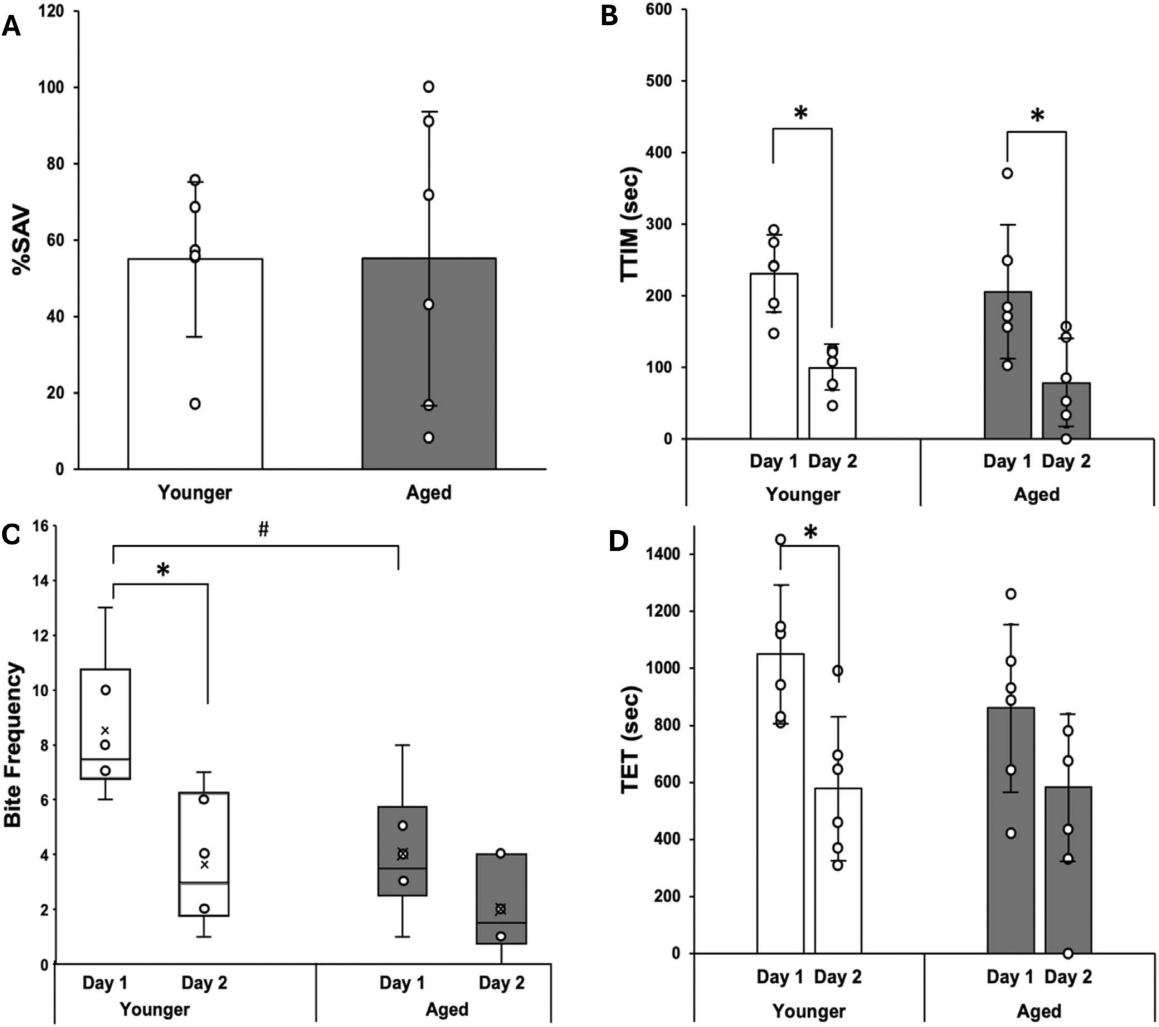
LFI metrics in CR sibling Aplysia. (A) Mean %SAV, ±SD (B) Mean TTIM, ±SD, on Day 1 and Day 2 training. *denotes significance difference via Wilcoxon Signed‐Rank; *p* ≤ 0.05 (younger *p* = 0.03125, aged *p* = 0.03125). (C) Distribution of bite frequency on Day 1 and Day 2 of LFI. *denotes significance difference via Wilcoxon Signed‐Rank test; *p* ≤ 0.05 (younger *p* = 0.03351). #denotes significance difference via multiple Wilcoxon Rank Sum tests and Bonferroni multiple test correction (Day 1 *p* = 0.02422). (D) Mean TET to completion of LFI for each day of training. *denotes significant difference via Paired t‐test; *p* ≤ 0.05 (younger *p* = 0.001069).

CR Aplysia, in contrast, did not show any difference in %SAV between TT1 and TT2 (*t*(7) = 0.01, *p* = 0.9918) (Figure 5A). A lone TT2 animal achieved 100% SAV, but the robust change in TTIM on Day 2 at both TT1 and TT2 (TT1: Wilcoxon, *p* ≤ 0.05; TT2: Wilcoxon, *p* ≤ 0.05) (Figure 5B) in the rest of the sample ensured no change in %SAV with age. When compared to their TT1 AL siblings, TT1 CR animals had a higher number of ingestion attempts, especially on Day 1 (Wilcoxon, *p* ≤ 0.05) (Figure 5C), a longer TTIM on Day 1, and a longer TET (Figure 5D), suggesting that TT1 CR animals were behaving hungrier than their AL fed siblings. The older CR animals sampled at TT2 did not display these behaviors.

### 3.5 Tail Withdrawal Reflex Habituation

**FIGURE 6:**
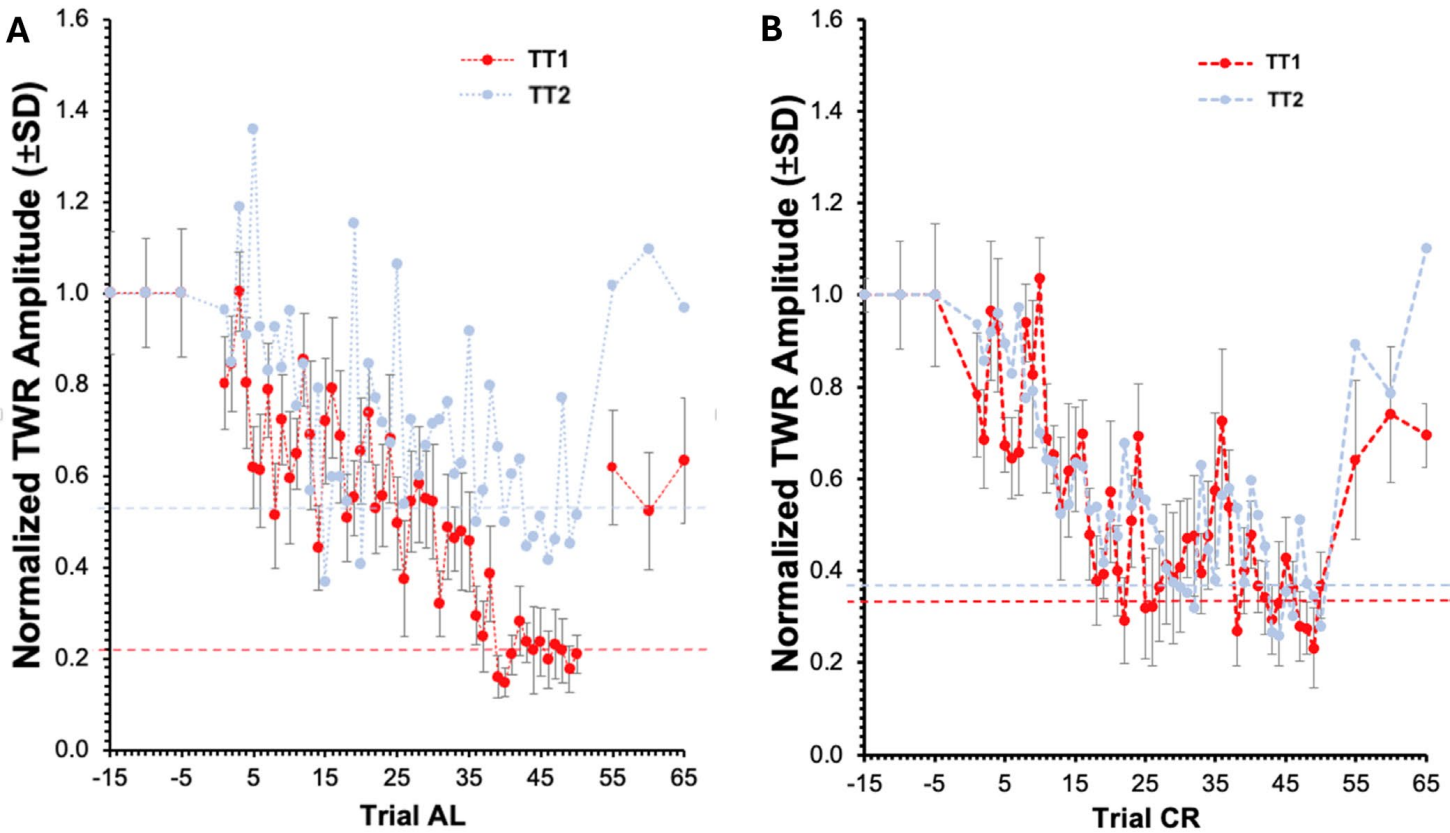
TWR habituation results for AL (A) and CR (B) animals that had +%SAV in LFI. Each point is the mean of 6 animals. Error bars of TT2 animals were removed for clearer visualization of the results. Dotted horizontal lines denote mean TWR amplitude of the last 10 trials of the corresponding color/shade group.

Since hunger may be a confounding variable in LFI, reflex habituation was measured as an independent test of aging effects on learning. Directly after Day 2 LFI training, animals were tested in habituation of their tail withdrawal reflex (TWR) (Figure 6). As expected, TT1 AL Aplysia habituated while TT2 AL Aplysia failed to habituate (Student's *T*‐test, *p* ≤ 0.01) (Figure 6A). However, all CR Aplysia tested at both TT1 and TT2 habituated their TWR (Figure 6B).

### 3.6 Gene Expression Results

Buccal ganglia samples were taken from all Aplysia that successfully displayed LTM in LFI. An equal number of buccal ganglia samples were taken from Aplysia that were not trained in LFI but reared under the same conditions as and sampled at the same time point as their trained counterparts. In total, 23 buccal ganglia samples from LFI trained Aplysia were sequenced along with 24 buccal ganglia from corresponding control Aplysia (Table 1).

**FIGURE 7:**
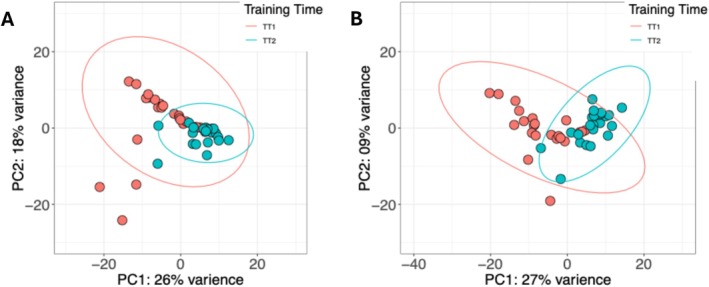
(A) Classical principal component analysis (PCA) colored by LFI training time points TT1 and TT2. Ellipses represent 99.9% confidence intervals (CI). The color of each ellipse corresponds to the training time point of the same color. (B) New PCA of 45 samples without outliers. Ellipses correspond to 99.9% CI for their respective training time point indicated by the matching colors of the samples.

Following summation to the gene level and removal of low count genes, there were 11,876 genes remaining across 47 samples. Principal component analysis (PCA) of variance stabilized transformed (VST) counts indicated tight clustering of samples according to their training time with principal component 1 (PC1) accounting for 26% of the variance in these data and PC2 accounting for 18% of the variance (Figure 7A). Upon further visual inspection of PCA, several samples fell outside their respective 95% confidence intervals (CIs). The CIs were then increased to 99.9% to bolster confidence in the visual identification of outlier samples, which still fell outside this increased CI. One TT2 sample and three TT1 samples fell outside their respective 99.9% CIs, classifying them as potential outliers. Robust principal component analysis (rPCA) was then run on the VST counts to statistically verify whether these samples were true outliers, and it was determined that two of the four visually classified outlier samples were true outliers (Figure S2). Both verified outlier samples were at training time TT1, LFI untrained, and diet CR. The outlier samples were removed and transcript abundances from the remaining samples were re‐imported into *R*, summarized to gene level, and filtered for the removal of low count genes. Outlier removal increased the number of genes available for downstream analyses to 12,314 genes across 45 samples.

Outlier removal also reduced the variance associated with PC2 by half. A second PCA was performed on VST count abundances of the remaining 45 samples (Figure 7B). This second PCA indicated PC1 accounting for 27% of the variance in these data, a negligible increase, and PC2 accounting for 9% of the variance. This noticeable decrease in the variance percentage value of PC2 suggests that the outlier samples were inflating this value in the original PCA. Overall, outlier removal increased the number of genes to be used in downstream functional analyses, which increased the ability to identify enriched terms and pathways, as well as resulted in a tighter and cleaner data set, as can be seen in the updated PCA.

**FIGURE 8:**
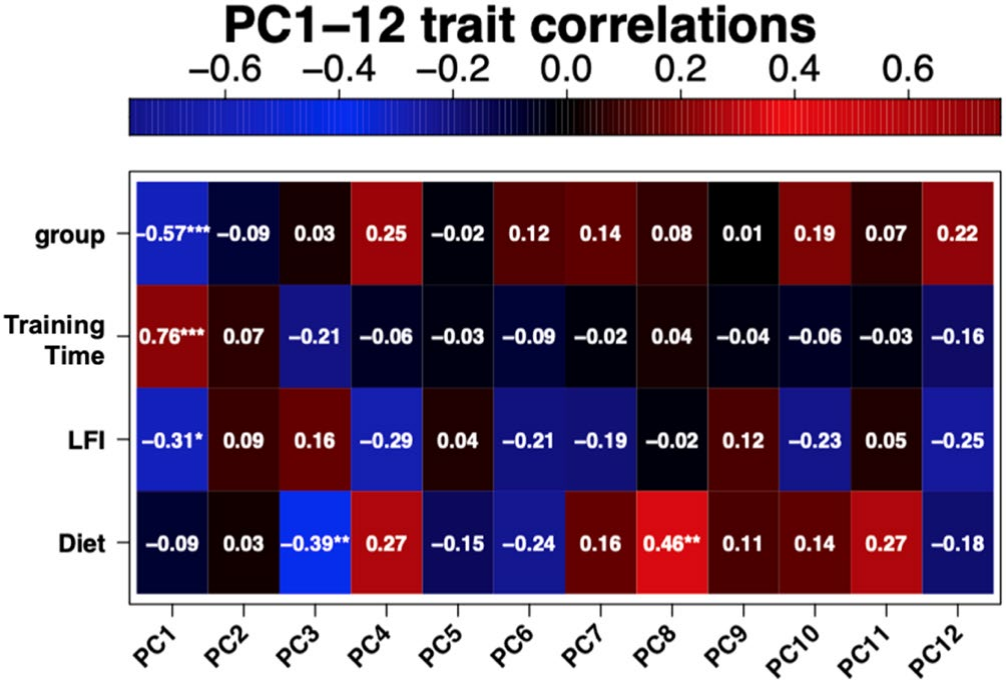
Heatmap of the Pearson correlation values of each PC's eigenvalue to the independent variables of this study. Red indicates a positive correlation while blue indicates a negative correlation. The exact Pearson correlation coefficients are displayed in each square. “*” indicates significance level with “*”, “**”, and “***” representing *p* ≤ 0.05, 0.01, and 0.001 respectively.

**FIGURE 9:**
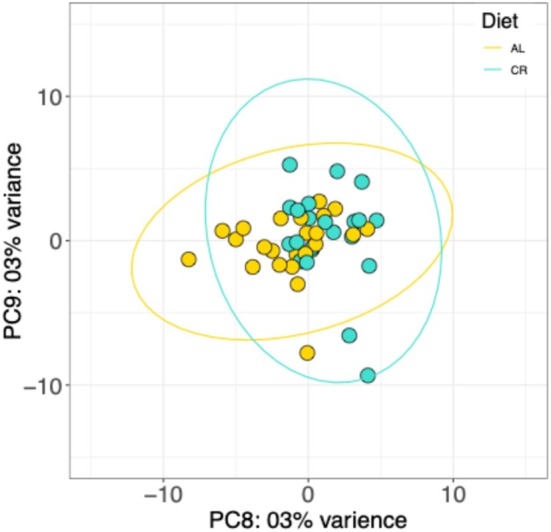
PCA colored for Diet. Ellipses are 99.9% CI and correspond to the respective colored samples and the diet associated with that color.

In an effort to identify drivers of the variance associated with each PC, eigenvalues that accounted for up to 70% of the variation in these data were correlated to each independent variable to identify any significant positive correlations. Negative correlations of eigenvalues to independent variables were not investigated, as a negative correlation would indicate that a variable's presence reduced the variance in these data for that PC, suggesting the associated variable was not a driver of variance but an inhibitor. Twelve eigenvalues (PC1—PC12) were necessary to account for 70% of the variance in these data (Figure S3). Pearson correlation results demonstrated training time was significantly positively correlated with PC1, indicating that the chronological age of the animals when tested in LFI was the largest driver of gene expression differences within the buccal ganglia microdissected samples of inter‐ and motoneurons (Figure 8). Diet was significantly positively correlated with PC8. A PCA plot of PC8/PC9 revealed that PC8 accounted for 3% of the variance in these data (Figure 9), which indicates that diet had a small, but significant, effect on gene expression variance. LFI was not significantly positively correlated to any of the PCs, which indicates that learning was not a strong enough driver to produce a detectable global gene expression difference in these samples.

Several contrasts were used to detect differentially expressed genes (DEGs) between conditions. Sensible nomenclature has been incorporated in their reporting (Table S1). For clarity, “Age AL” refers to chronologically older TT2UAL vs. chronologically younger TT1UAL, “Age CR” refers to TT2UCR vs. TT1UCR, “LFI young AL” refers to TT1TAL vs. TT1UAL, “LFI aged AL” refers to TT2TAL vs. TT2UAL, “LFI young CR” refers to TT1TCR vs. TT1UCR, and “LFI aged CR” refers to TT2TCR vs. TT2UCR. Each contrast relates to how genes or functional analysis terms change in the first group compared to the second.

**FIGURE 10:**
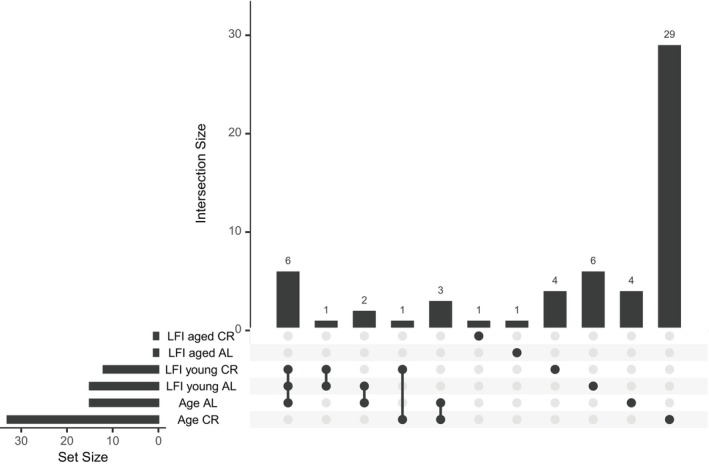
All 58 Differentially expressed genes for the six contrasts of the eight total interactions (group). Set Size indicates the DEGs that were contributed by that comparison. Dark nodes next to a comparison indicate that it contributed to the Intersection Size above. Where multiple nodes are filled in, multiple comparisons shared a DEG. The number of DEGs either shared by multiple comparisons or unique to a particular comparison is indicated by the number on top of the Intersection Size bars.

**FIGURE 11:**
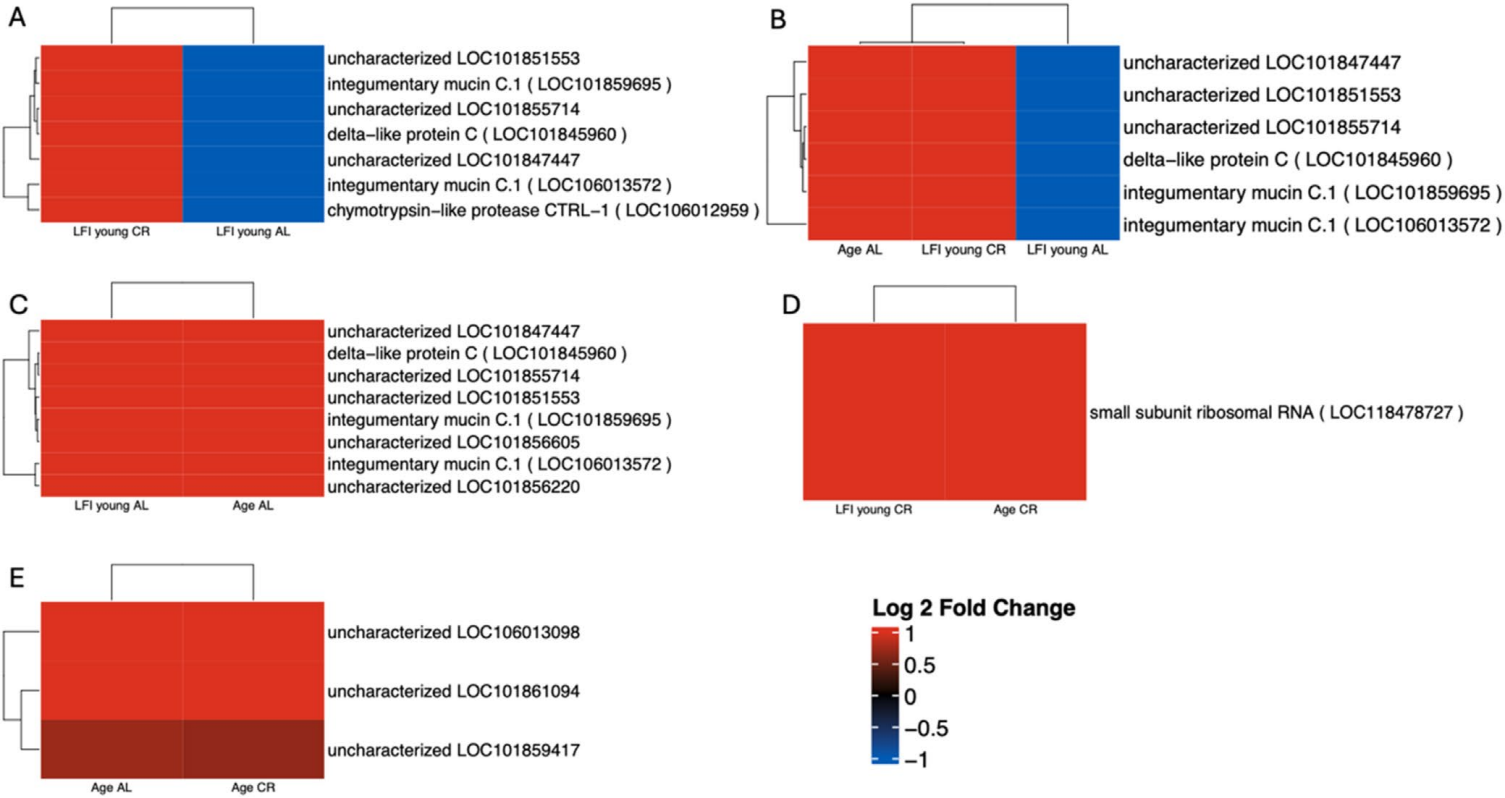
Heatmaps indicate the log fold change (LFC) of DEGs that were present in more than one contrast. Red reflects upregulated while green reflects downregulated.

Few genes were differentially expressed in each of the six contrasts, yet there were some DEGs that were present in more than one of the DESeq2 contrasts run. Not all were regulated in the same fashion. DEGs that appeared in more than one contrast run are referred to as shared DEGs. The full list of DEGs from each contrast is in File S1 and a graphical representation of the total number of DEGs as well as the number of shared DEGs is illustrated in Figure 10. Age CR animals showed the largest gene expression difference. Many of the shared DEGs were uncharacterized or mucin genes. Of note are seven shared DEGs between LFI young AL and LFI young CR animals because of their opposing regulation. These seven genes were downregulated after LFI recall in younger AL animals, and upregulated in younger CR animals (Figure 11A). Three of the seven genes were uncharacterized, two were mucin genes, one was a delta‐like protein C, and one was a chymotrypsin‐like protease CTRL‐1. Six of these seven genes, all except chymotrypsin‐like protease CTRL‐1, were also upregulated in Age AL animals (Figure 11B). Other shared DEGs present in other contrasts were regulated in the same fashion (Figure 11C–E).

**FIGURE 12:**
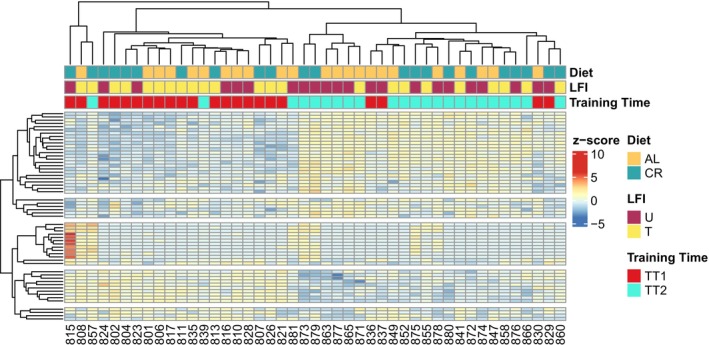
Hierarchical heat map of all DEGs from group comparisons. Each column is a sample. Each sample's associated independent variables are indicated by the colors of the row tiles within the three rows above the heat map. Colors within the heatmap indicate the *z*‐score of a DEG where red indicates that gene was expressed more than its mean in all samples (upregulated) and blue indicates that gene was expressed less than its mean (downregulated).

Hierarchical clustering of all DEGs from the six contrasts revealed samples clustered most tightly according to their training time (chronological age), then diet, and finally LFI status (Figure 12). These results support the PCA results in suggesting training time was the largest determinant in gene expression differences in the samples, followed by diet.

**FIGURE 13:**
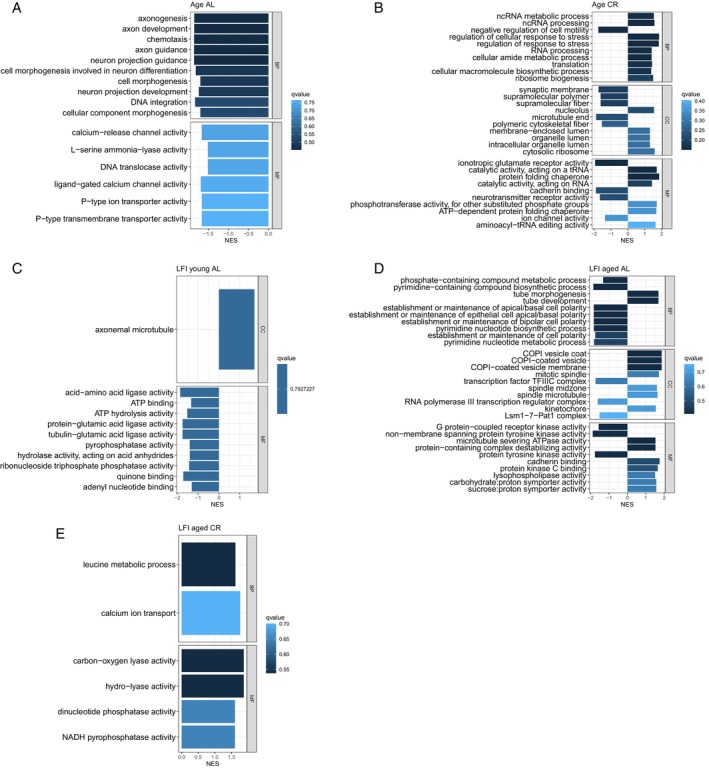
Ten most significantly enriched GO terms within the three ontologies for each contrast except LFI young CR. NES = normalized enrichment score.

**FIGURE 14:**
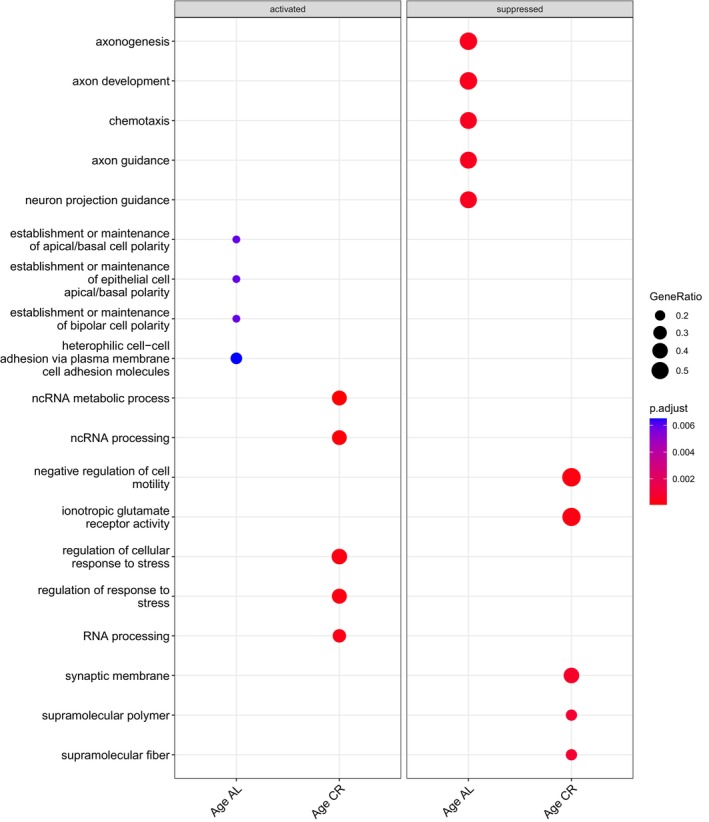
GSEA results for the five most significantly activated and suppressed enriched GO terms in Age AL compared with Age CR.

**FIGURE 15:**
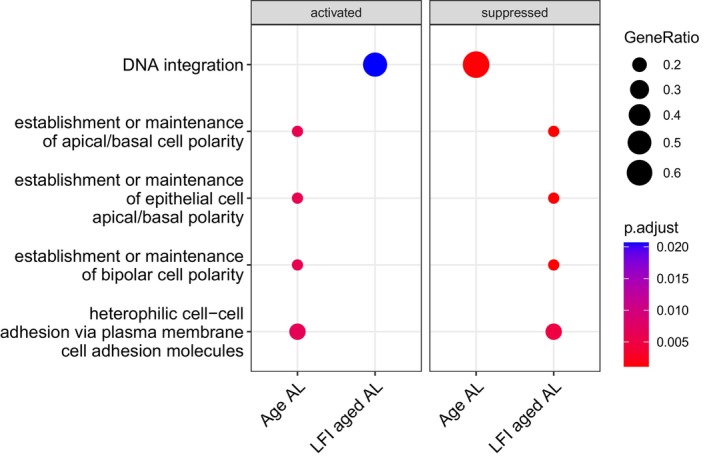
GSEA results for the 5 enriched GO terms shared between Age AL and LFI aged AL, demonstrating their opposite regulation. Symbol size indicates the gene ratio and color the significance.

Gene set enrichment analysis (GSEA) was performed on each gene set from the six contrasts to identify significantly enriched gene ontology (GO) terms. There were 889 significantly enriched GO terms in total for all contrasts. After qvalue filtering, there were none present in LFI young CR. A full list of significantly enriched GO terms for each contrast is in File S2. The 10 most significantly enriched GO terms within each of the three GO ontologies were plotted for each contrast (Figure 13A–E). Age AL and Age CR did not share terms in their 10 most significantly enriched GO terms (Figure 14). Of note are *microtubule severing ATPase activity* (GO:0008568), *cadherin binding* (GO:0045296), and *protein kinase C binding* (GO:0005080); three GO terms that were significantly enriched in LFI aged AL and not in Age AL. There were also four significantly enriched GO terms of the biological process ontology that were suppressed in LFI age AL, which were also significantly enriched, yet activated, in Age AL (Figure 15). Two of the four GO terms just mentioned were *establishment or maintenance of bipolar cell polarity* (GO:0061245) and *heterophilic cell–cell adhesion* via *membrane‐bound cell adhesion molecules* (GO:0007157).

**FIGURE 16:**
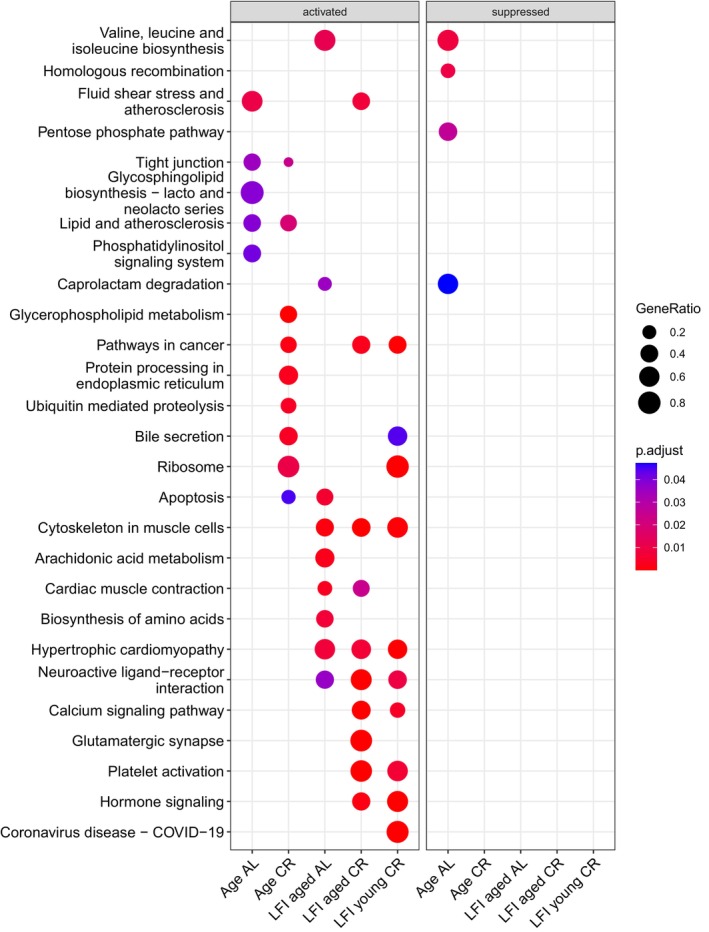
GSEA results for the 5 most significant KEGG pathways for each comparison except LFI young AL (no significant pathways), demonstrating presence or absence of significantly enriched terms between comparisons. There were no suppressed pathways in any comparisons except Age AL. Symbol size indicates the gene ratio and color the significance.

GSEA was also performed on each gene set from the six contrasts to identify significantly enriched Kyoto Encyclopedia of Genes and Genomes (KEGG) pathways. There were 176 significantly enriched KEGG pathways in total for all contrasts. After *q* value filtering there were none present in LFI young AL. A full list of significantly enriched KEGG pathways for each contrast is in File S2. The five most significantly enriched KEGG pathways were plotted for each contrast (Figure 16). Of interest are the pathways *cytoskeleton in muscle cells* (ko04820) and *neuroactive ligand‐receptor interaction* (ko04080), which were significantly enriched and activated in LFI trained animals only.

**FIGURE 17:**
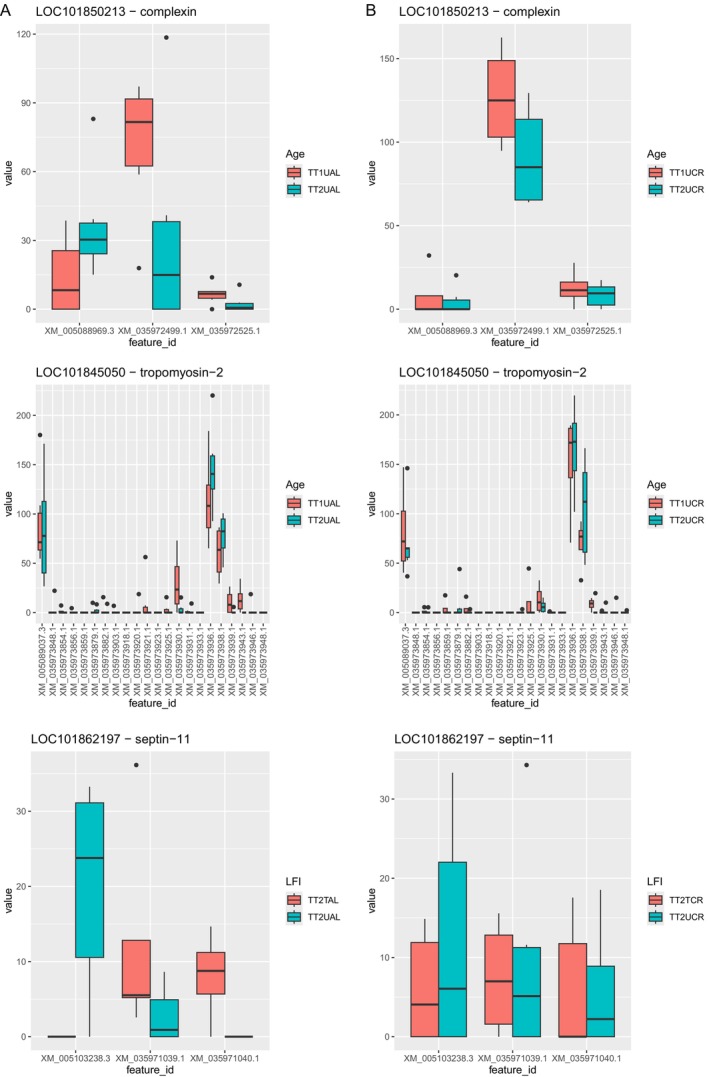
The expression value of each transcript within a notated gene is plotted to compare significant DTU in Age AL and LFI aged AL (A) to non‐significant DTU in Age CR and LFI aged CR (B).

Finally, differential transcript usage (DTU) was investigated to infer isoform switching or alternative splicing events that may be occurring within contrasts. There were two genes in Age AL and one gene in LFI aged AL for which there was significant evidence of DTU. The genes *complexin* and *tropomyosin‐2* were found to participate in DTU in Age AL and the gene *septin‐11* underwent DTU after learning in LFI aged AL (Figure 17A). These same genes did not demonstrate significant DTU in Age CR or LFI aged CR (Figure 17B).

## 4 Discussion

Calorie restriction (CR) did not extend the lifespan of *Aplysia californica* (Aplysia) compared to siblings reared on ad‐lib (AL). This result was surprising as life extending properties of CR have been reported for decades, for example, in rats fed approximately 40% reduced caloric intake compared to their AL fed counterparts [4]. Since then, CR has been shown to extend the life of many other model species when calories are restricted by 20%–30% [78]. Recently, significant life extension has been observed in rats on as little as a 10% CR diet [79]. Meanwhile, in humans, CR is often defined as a 25% reduction in calories needed for Total Daily Energy Expenditure (TDEE; [80]). CR has not been stringently defined in Aplysia. In practical terms, the optimal food intake for healthy Aplysia at the National Aplysia Resource is 90% total body mass over 6 days [47]. Therefore, in relation to humans, the current diet at the National Aplysia Resource covers Aplysia's TDEE. An additional −25% yielded the 65% CR diet used in this study. Several other studies conducted in our laboratory have addressed the effects of CR on lifespan in Aplysia with conflicting results [47, 81, 82, 83]. Taken together, this suggests that despite differences in the constituents of the diet and the duration of the feeding regimens in these studies, the lifespan of Aplysia appears more dependent on the cohort (genetics) than on diet.

Another explanation for the lack of life extension in CR Aplysia may be the ambient conditions of their habitat. *Aplysia californica* are naturally found in the cool shores of California, mimicked by housing at 15°C at the National Aplysia Resource. While animals that regulate their body temperature will increase their metabolic rate in cooler temperatures, the reverse is true for ectotherms, where cooler temperatures generally result in a reduction of their metabolic rate. Since one mechanism by which CR is proposed to extend lifespan is by reducing the metabolic rate of the organism [84], an already reduced metabolic rate due to temperature may not be able to be reduced further by CR, thus limiting CR's beneficial effects. *Drosophila melanogaster* reared at 21°C–23°C did not show life extension on a CR diet compared to their counterparts reared at ambient temperature [85]. Thus, body temperature may have a greater effect on lifespan than metabolic rate changes due to CR [86].

Although lifespan extension was not observed, a delay in biological aging was. That CR delays biological aging at all is a controversial idea and highly dependent on the way in which biological aging is measured. For example, the CALERIE study noted a delay in biological aging in participants who followed a 25% reduced calorie diet over 2 years when the data were analyzed for homeostatic dysregulation and the Klemera‐Doubal Method for Biological Age [87]. Yet, when these same data were analyzed against DNA methylation aging clocks such as PhenoAge and GrimAge, there were no indications that CR delayed biological aging [88]. In our model, CR appears to have delayed aging by 3 measures: 2 reflexes, 2 distinct tests of learning, and the gene expression results.

Since we had previously reported that aged AII AL Aplysia did not habituate their tail withdraw reflex (TWR) [55], failure of the AII AL animals to habituate in this study was not unexpected. Supportive of the beneficial effects of CR, however, both old and younger CR animals habituated. This suggests that CR delayed progression into advanced biological age as assessed by both reflex execution and habituation of a reflex.

Similarly, analysis of CR Aplysia that demonstrated a long‐term memory (LTM) of learning food is inedible (LFI) showed performance was unaffected by chronological age. While CR did not improve learning performance as in mammals [35, 38, 89, 90], it corresponded to sustained learning performance in age. In contrast, this is the second study from our laboratory to show that aged AL Aplysia had a superior recall of LFI LTM than their younger siblings [51], even though they failed at habituation. It is possible that tail withdrawal and buccal feeding motor patterns age at different rates, as demonstrated by Peretz et al. [91], Zolman and Peretz [92], and proposed by Moroz and Kohn [93]. Alternatively, daily use of the feeding circuit as the animal ages may mitigate any of the neuronal impairments, as proposed by Swaab [94], whereas TWR behavior may not be called upon as frequently in a controlled environment.

Reference [55] determined that the failure to habituate TWR in aged Aplysia was associated with underlying neuronal impairment of the circuit controlling the reflex. Therefore, it is plausible that CR mitigated senescence in the neural circuit controlling TWR based on the behavioral results observed here. This is speculative as the neural circuit controlling TWR was not investigated in this study. Additionally, it is important to note that since the circuit of TWR was not assayed for transcription, the existing transcriptional results are specific to the circuitry of LFI and thus should not be interpreted as involving the circuitry of TWR.

Finally, when comparing gene expression results from the contrasts of Age AL and Age CR animals, differences in their biological age on the transcriptional level appeared. This was surprising as CR accounted for only 3% of the total variance in these data, yet several significant genes and gene ontology (GO) terms appear to reflect CR conditions. One example is the presence of the significantly differentially expressed gene (DEG) heat shock protein 70 (*Hsp70*) in Age CR but not in Age AL. *Hsp70* upregulation is common in older animals, which appears to reflect a compensatory mechanism to the loss of proteostasis, a known aging hallmark in many species, including Aplysia [95, 96]. Its absence in Age AL is perplexing, but its upregulation in both Age CR and LFI mature CR is in line with its reported behavior in other CR studies [97], although its absence in LFI aged CR is also interesting. It is hypothesized that the complexity of the current study's design resulted in multiple test corrections, which potentially yielded a severely limited number of DEGs and could explain some of the inconsistencies of their presence or absence in the multiple contrasts, culminating in contradictory patterns. Still, several other DEGs in Age CR increased as expected, such as eukaryotic translation initiation factors (eIF) 5, 5A‐1, and 2 subunit 2 [98, 99]. These suggest altered protein synthesis in CR animals, potentially limiting those proteins needed for growth and increasing those with neuronal protective properties. The upregulation of proteosome subunit alpha type‐1 [100, 101] reflects an altered proteosome system and protein recycling. There was also an upregulation of MLX‐interacting protein [102], which is involved in regulating metabolism in response to glucose levels. It is important to note that although these DEGs were present in Age CR, they were not significantly differentially expressed in LFI mature CR or LFI aged CR samples, further highlighting the contradictory patterns of DEGs between multiple contrasts. Other examples of potential transcriptional responses to CR were the downregulation of multiple genes within significantly enriched gene ontology (GO) terms of Age AL animals, which resulted in the gene set enrichment analysis (GSEA) deeming these GO terms suppressed in age. Many of these suppressed GO terms in Age AL were directly related to neuron development, such as *axonogenesis* (GO:0007409), *axon development* (GO:0061564), *chemotaxis* (GO:0006935), *axon guidance* (GO:0007411), and *neuron projection guidance* (GO:0097485). Age AL animals also showed significant enrichment in multiple GO terms related to the reduction in cellular differentiation, which the GSEA showed as activated in age. One set of related activated GO terms in Age AL animals were *establishment or maintenance of apical/basal cell polarity* (GO:0035088), *establishment or maintenance of epithelial cell apical/basal polarity* (GO:0045197), *establishment or maintenance of bipolar cell polarity* (GO:0061245), and *establishment or maintenance of cell polarity* (GO:0007163). Another set of related activated GO terms in Age AL animals was *heterophilic cell–cell adhesion* via *plasma membrane cell adhesion molecules* (GO:0007157) and *cell–cell adhesion* via *plasma‐membrane adhesion molecules* (GO:0098742). These sets of related activated GO terms indicate an increased effort to maintain buccal neuronal polarity in aged AL Aplysia, a process that has also been shown in cerebellar neurons of aging mice [103] and pleural ventral caudal neurons of Aplysia [96]. CR appears to have played a role in mitigating these age‐related transcriptional responses since none of these GO terms were significantly enriched in Age CR animals. Instead, Age CR animals showed significant enrichment of GO terms activated in GSEA that related to RNA processing and the response to stress. The absence of significantly enriched GO terms in aged neurons of CR Aplysia, which are commonly seen in aged neurons of AL animals, might point to a delay in their onset. More studies are necessary, due to the low variance associated with CR from the PCA reported, to confirm that these lines of evidence were induced by CR and not some other condition.

Several related lines of evidence also suggest synaptic remodeling after recall of LFI and an association between remodeling and increased learning performance. Many genes implicit in learning, memory, and neuronal functioning were differentially expressed in age, such as the downregulation of cyclic nucleotide‐gated olfactory channel [104] and serine/threonine‐protein kinase PAR‐1 [105] in Age AL, which are involved in olfactory transduction as well as neuronal polarity and dendritic spine formation, respectively. Calbindin‐32 [106] and pannexin 10 [107], which are involved in modulating neuronal excitability through regulating calcium levels and ATP release, respectively, were downregulated in Age CR, which is consistent with aged CR animals not performing as well as aged AL animals in LFI. Finally, TAR DNA‐binding protein 43 [108], whose deregulation is linked to cognitive decline and neurodegenerative diseases, as well as major vault protein [109], which has been implicated in the regulation of experience‐dependent plasticity, were both upregulated in Age CR. Yet these DEGs were only present in aged controls of either diet and were not significantly different in animals that learned in LFI. Therefore, the ability to draw conclusions based on these DEGs is limited. Therefore, it is instructive to focus on the results of the GSEA beginning with the significantly enriched GO terms that were activated in LFI aged AL animals included *microtubule severing ATPase activity* (GO:0008568), *cadherin binding* (GO:0045296), and *protein kinase C binding* (GO:0005080), which may be aiding in the proposed active reduction in neuronal processes after LFI recall, which would effectively strengthen the LTM of LFI. These GO terms suggest microtubules that structurally support synapses are being severed and rebuilt elsewhere. This process may be facilitated by protein kinase C, a known regulator of synaptic plasticity [110]. This proposed mechanism is similar to how long‐term habituation in Aplysia is associated with the reduction of sensory to motoneuron presynaptic terminals and branches [111], an occurrence which is also observed after LFI between sensory to moto‐ and interneurons [54]. The buccal ganglia samples in this study were microdissected to eliminate the sensory neurons, therefore these signatures may be coming from interneurons. There is evidence that excitatory interneurons show signs of depression during habituation [112], and while depression does not necessarily reflect a change in terminal morphology, its presence along with other forms of plasticity in interneurons strongly suggests their importance in memory retention as previously reviewed in Aplysia [113]. Therefore, the depression of inter‐ or motoneuronal synapses, especially if they were associated with the protraction of the radula bulb during the feeding reflex, such as those from B31/B32 onto the I2 nerve, would reduce the likelihood of protraction occurring during tactile lip stimulation. Such a mechanism would result in an end to attempts to ingest the probe during LFI training. In addition, the significantly enriched pathway *valine, leucine and isoleucine biosynthesis* (ko00290) was activated in LFI aged AL animals. This reflects an increase in branched‐chain amino acids (BCAA), which have been linked to higher learning capabilities in rats and an increase in acetylcholine synthesis [114]. *Valine, leucine and isoleucine biosynthesis* (ko00290) was suppressed in Age AL, further suggesting that LFI is driving these transcriptional changes. Since LFI's status in controlling transcription is speculative, morphological or electrophysiological assays would be needed to validate these hypotheses.

DTU analysis suggests isoform switching occurs as AL animals advance in chronological age as well as after LFI. Among these, *complexin* is a known regulator of vesicle exocytosis [115] and its difference of transcript usage in age may reflect differences in neurotransmitter release. Interestingly, there was no evidence of *complexin* DTU in Age CR. *Tropomyosin‐2* regulates actin‐associated proteins, facilitates actin filament stabilization, and aids in synaptic neurotransmitter receptor trafficking [116]. Its DTU in Age AL suggests a change in these functions associated with age that was not observed in Age CR. Finally, *septin‐11* shows evidence of total DTU between control TT2 AL animals and those trained in LFI, suggesting an isoform switch after learning. *Septin‐11* plays a role in neuronal cytoarchitecture and is highly associated with gamma aminobutyric acid (GABA)ergic synapses [117]. The coordinated effort of certain buccal ganglia GABAergic inhibitory interneurons has previously been shown to contribute to the ingestive function of the Aplysia feeding motor network [118]. Thus, the switching of *septin‐11* isoforms after LFI in TT2 AL animals may indicate a change in GABAergic synapses, resulting in a change of the feeding motor network leading to fewer ingestion attempts during LFI and better learning overall. Again, this was not observed in LFI aged CR animals, coincident with their poorer performance compared to their AL siblings. Direct functional assays might reveal the impact of DTU in age and LFI.

More research, such as functional validation studies involving the knockdown or overexpression of these transcriptional alterations, is needed to elucidate the role transcriptional changes play in behavioral performance during learning. Still, these results demonstrate the ways that expression of specific genes in sibling groups change with aging at different rates when diet and learning are superimposed. Although these genes have numerous functions across the physiological landscape of aging, their patterns in this model are examples of expression potentially relevant in other learning archetypes.

## Funding

This work was funded by the National Institutes of Health Grant (P40OD010952). Manuscript preparation was partially funded in association with the National Science Foundation Grant (2213824).

## Conflicts of Interest

The authors declare no conflicts of interest.

## Supporting information


**Figure S1:** The table represents the 2 × 2 × 2 multifactorial design of the study. Below the table shows how the model matrix was designed using full interaction terms. All interaction terms are then listed.
**Figure S2:** Results from the robust principal component analysis (rPCA) performed using PcaGrid from the rrcove package in *R*. Two samples fell above the line demarcating statistically significant outliers and were removed from the dataset for downstream analyses.
**Figure S3:** SCREE plot showing the amount of variance each PC accounts for. Greater than 70% of the variance in these data is accounted for by PC12.

**Table S1:** The six different contrasts and the nomenclature used to describe them in the text.
**Table S2:** Proximate analysis of Aplysia foot muscle tissue from the two diet groups of ad lib feeding (AL) and calorie restriction (CR).
**Table S3:** Fatty acid profile of Aplysia foot muscle tissue from the two diet groups.
**Table S4:** Total fatty acid profile of Aplysia foot muscle tissue from the two diet groups.
**Table S5:** Total omega fatty acid profile of Aplysia foot muscle tissue from the two diet groups.

**File S1:** gbb70046‐sup‐0003‐SupplementaryFile1.xlsx.

**File S2:** gbb70046‐sup‐0004‐SupplementaryFile2.xlsx.

**Data S1:** gbb70046‐sup‐0005‐supinfo.docx.

## Data Availability

The data that support the findings of this study are openly available in Sequence Read Archive at https://www.ncbi.nlm.nih.gov/bioproject/1290641, reference number PRJNA1290641.
