# Supplementary material for: Calorie Restriction Effects on Aging, Learning Performance, and Transcription in Aged Aplysia californica
Source: Genes Brain Behav. 2026 Feb 26;25(1):e70046. doi: 10.1111/gbb.70046 (PMC12937505; doi:10.1111/gbb.70046)
Supplement: Supplementary file 1 — Figure S1: The table represents the 2 × 2 × 2 multifactorial design of the study. Below the table shows how the model matrix was designed using full interaction terms. All interaction terms are then listed. Figure S2: Results from the robust principal component analysis (rPCA) performed using PcaGrid from the rrcove package in R. Two samples fell above the line demarcating statistically significant outliers and were removed from the dataset for downstream analyses. Figure S3: SCREE plot showing the amount of variance each PC accounts for. Greater than 70% of the variance in these data is accounted for by PC12. [file GBB-25-e70046-s005.docx]

|  |
| --- |
| **Supplemental Figure 1.** |

|  |
| --- |
| **Supplemental Figure 2.** |

|  |
| --- |
| **Supplemental Figure 3.** |

**Supplemental Figure 1.** The table represents the 2x2x2 multifactorial design of the study. Below the table shows how the model matrix was designed using full interaction terms. All interaction terms are then listed.

**Supplemental Figure 2** Results from the robust principal component analysis (rPCA) performed using PcaGrid from the rrcove package in R. Two samples fell above the line demarcating statistically significant outliers and were removed from the dataset for downstream analyses.

**Supplemental Figure 3.** SCREE plot showing the amount of variance each PC accounts for. Greater than 70% of the variance in these data is accounted for by PC12.
