# Supplementary material for: Calorie Restriction Effects on Aging, Learning Performance, and Transcription in Aged Aplysia californica
Source: Genes Brain Behav. 2026 Feb 26;25(1):e70046. doi: 10.1111/gbb.70046 (PMC12937505; doi:10.1111/gbb.70046)
Supplement: Supplementary file 2 — Table S1: The six different contrasts and the nomenclature used to describe them in the text. Table S2: Proximate analysis of Aplysia foot muscle tissue from the two diet groups of ad lib feeding (AL) and calorie restriction (CR). Table S3: Fatty acid profile of Aplysia foot muscle tissue from the two diet groups. Table S4: Total fatty acid profile of Aplysia foot muscle tissue from the two diet groups. Table S5: Total omega fatty acid profile of Aplysia foot muscle tissue from the two diet groups. [file GBB-25-e70046-s004.docx]

**Supplemental Table 1.** The six different contrasts and the nomenclature used to describe them in the text.

| **Nomenclature** | **Contrast** | **Description** | **Definitions** |
| --- | --- | --- | --- |
| Age AL | TT2UAL vs TT1UAL | Gene expression differences influenced by age in AL animals | TT1 = chronologically younger animals  TT2 = chronologically older animals  U = untrained in LFI  T = trained in LFI  AL = reared on AL diet  CR = reared on CR diet |
| Age CR | TT2UCR vs TT1UCR | Gene expression differences influenced by age in CR animals |  |
| LFI young AL | TT1TAL vs TT1UAL | Gene expression differences influenced by LFI in younger AL animals |  |
| LFI aged AL | TT2TAL vs TT2UAL | Gene expression differences influenced by LFI in older AL animals |  |
| LFI young CR | TT1TCR vs TT1UCR | Gene expression differences influenced to by LFI in younger CR animals |  |
| LFI aged CR | TT2TCR vs TT2UCR | Gene expression differences influenced by LFI in older CR animals |  |

**Supplemental Table 2.** Proximate analysis of Aplysia foot muscle tissue from the two diet groups of ad lib feeding (AL) and calorie restriction (CR).

| **Proximate Analysis** |  |  |  |
| --- | --- | --- | --- |
|  | **AL** | **CR** | **Difference** |
| **% Moisture** | 85.9 ± 1.27 | 85.9 | 0 |
| **% Protein** | 10.45 ± 1.06 | 10.6 | 0.15 |
| **% Fat** | 0.35 ± 0.07 | 0.7 | 0.35 |
| **% Ash** | 2.7 ± 0.14 | 2.6 | 0.1 |
| **% Carbohydrates** | 0.6 ± 0.14 | 0.2 | 0.4 |
|  |  | **Average** | 0.2 |

*AL data were expressed as the mean ± SD of two samples. There were only one sample for CR data.*

**Supplemental Table 3.** Fatty acid profile of Aplysia foot muscle tissue from the two diet groups.

| **Fatty Acid Profile** |  |  |  |
| --- | --- | --- | --- |
|  | **AL** | **CR** | **Difference** |
| **% Butyric (C4:0)** | <0.01 | <0.01 | 0.00 |
| **% Caproic (C6:0)** | <0.01 | <0.01 | 0.00 |
| **% Caprylic (C8:0)** | 0.31 | 0.26 | 0.05 |
| **% Capric (C10:0)** | <0.01 | <0.01 | 0.00 |
| **% Lauric (C12:0)** | 0.30 ± 0.24 | 0.31 | 0.01 |
| **% Tridecanoic (C13:0)** | 0.38 ± 0.12 | 0.46 | 0.09 |
| **% Myristic (C14:0)** | 0.84 ± 0.06 | 0.71 | 0.13 |
| **% Myristoledic (C14:1 Trans)** | <0.01 | <0.01 | 0.00 |
| **% Myristoleic (C14:1 Cis)** | <0.01 | <0.01 | 0.00 |
| **% Pentadecanoic (C15:0)** | 0.36 | 0.28 | 0.08 |
| **% Palmitic (C16:0)** | 11.40 ± 0.28 | 10.30 | 1.10 |
| **% Palmitelaidic (C16:1 Trans)** | <0.01 | <0.01 | 0.00 |
| **% Palmitoleic (C16:1 Cis)** | 1.24 ± 0.11 | 1.15 | 0.09 |
| **% Heptadecanoic (C17:0)** | 0.74 ± 0.16 | 0.64 | 0.10 |
| **% 10-Heptadecanoic (C17:1)** | 6.65 ± 8.56 | 13.80 | 7.16 |
| **% Stearic (C18:0)** | 13.75 ± 0.92 | 12.60 | 1.15 |
| **% Eliadic (C18:1 Trans)** | 0.21 | <0.01 | 0.21 |
| **% Oleic (C18:1 Cis)** | 15.55 ± 1.63 | 15.30 | 0.25 |
| **% Linolelaidic (C18:2 Trans)** | <0.01 | <0.01 | 0.00 |
| **% Linoleic (C18:2 Cis)** | 6.54 ± 1.63 | 6.71 | 0.18 |
| **% gamma-Linolenic (C18:3 gamma)** | <0.01 | <0.01 | 0.00 |
| **% Nonadecanoic (C19:0)** | <0.01 | <0.01 | 0.00 |
| **% alpha-Linolenic (C18:3 alpha)** | 2.66 ± 1.12 | 1.72 | 0.94 |
| **% Arachidic (C20:0)** | <0.01 | <0.01 | 0.00 |
| **% 11-Eicosenoic (C20:1)** | 1.36 ± 0.13 | 1.16 | 0.20 |
| **% 11-14 Eicosadienoic (C20:2)** | 2.80 ± 0.13 | 2.32 | 0.48 |
| **% Homo-gamma linolenic (C20:3)** | 1.52 ± 0.14 | 1.29 | 0.23 |
| **% 11-14-17 Eicosatrienoic (C20:3)** | 1.13 | 0.91 | 0.22 |
| **% Arachidonic (C20:4)** | 26.15 ± 2.33 | 23.80 | 2.35 |
| **% Eicosapentaenoic (C20:5)** | 4.90 ± 0.38 | 4.66 | 0.24 |
| **% Heneicosanoic (C21:0)** | <0.01 | <0.01 | 0.00 |
| **% Behenic (C22:0)** | <0.01 | <0.01 | 0.00 |
| **% Erucic (C22:1)** | <0.01 | <0.01 | 0.00 |
| **% Docosadienoic (C22:2)** | <0.01 | <0.01 | 0.00 |
| **% Docosapentaenoic (C22:5)** | 2.21 ± 0.86 | 1.55 | 0.66 |
| **% Docosahexaenoic (C22:6)** | <0.01 | <0.01 | 0.00 |
| **% Tricosanoic (C23:0)** | <0.01 | <0.01 | 0.00 |
| **% Lignoceric (C24:0)** | <0.01 | <0.01 | 0.00 |
| **% Nervonic (C24:1)** | <0.01 | <0.01 | 0.00 |
|  |  | **Average** | 0.41 |

*AL data were expressed as the mean ± SD of two samples when applicable. There were only one sample for CR data.*

**Supplemental Table 4.** Total fatty acid profile of Aplysia foot muscle tissue from the two diet groups.

| **Total Fatty Acids** |  |  |  |
| --- | --- | --- | --- |
|  | **AL** | **CR** | **Difference** |
| **% Saturated fat** | 27.7 ± 0.99 | 25.6 | 2.1 |
| **% Polyunsaturated fats** | 47.4 ± 6.08 | 43 | 4.4 |
| **% Monounsaturated fats** | 24.8 ± 7.21 | 31.4 | 6.6 |
| **% Trans fatty acids** | 0.21 | <0.01 | 0.21 |
|  |  | **Average** | 3.33 |

*AL data were expressed as the mean ± SD of two samples when applicable. There were only one sample for CR data.*

**Supplemental Table 5.** Total omega fatty acid profile of Aplysia foot muscle tissue from the two diet groups.

| **Total Omega Fatty Acids** |  |  |  |
| --- | --- | --- | --- |
|  | **AL** | **CR** | **Difference** |
| **% Omega 3 fatty acids** | 10.35 ± 3.18 | 8.84 | 1.51 |
| **% Omega 6 fatty acids** | 37.05 ± 2.90 | 34.2 | 2.85 |
| **% Omega 9 fatty acids** | 15.55 ± 1.63 | 15.3 | 0.25 |
|  |  | **Average** | 1.54 |

*AL data were expressed as the mean ± SD of two samples when applicable. There were only one sample for CR data.*
